# The Inhibitory Effects of Anti-Oxidants on Ultraviolet-Induced Up-Regulation of the Wrinkling-Inducing Enzyme Neutral Endopeptidase in Human Fibroblasts

**DOI:** 10.1371/journal.pone.0161580

**Published:** 2016-09-20

**Authors:** Hiroaki Nakajima, Shuko Terazawa, Takao Niwano, Yorihiro Yamamoto, Genji Imokawa

**Affiliations:** 1 Toyo Beauty Co. Ltd., R&D Division, Osaka, Japan; 2 School of Bioscience and Biotechnology, Tokyo University of Technology, Tokyo, Japan; 3 Research Institute for Biological Functions, Chubu University, Aichi, Japan; University of Alabama at Birmingham, UNITED STATES

## Abstract

We recently reported that the over-expression of skin fibroblast-derived neutral endopeptidase (NEP) plays a pivotal role in impairing the three-dimensional architecture of dermal elastic fibers during the biological mechanism of ultraviolet (UV)-induced skin wrinkling. In that process, a UVB-associated epithelial-mesenchymal cytokine interaction as well as a direct UVA-induced cellular stimulation are associated with the up-regulation of NEP in human fibroblasts. In this study, we characterized the mode of action of ubiquinol_10_ which may abrogate the up-regulation of NEP by dermal fibroblasts, resulting in a reported in vivo anti-wrinkling action, and compared that with 3 other anti-oxidants, astaxanthin (AX), riboflavin (RF) and flavin mononucleotide (FMN). Post-irradiation treatment with all 4 of those anti-oxidants elicited an interrupting effect on the UVB-associated epithelial-mesenchymal cytokine interaction leading to the up-regulation of NEP in human fibroblasts but with different modes of action. While AX mainly served as an inhibitor of the secretion of wrinkle-inducing cytokines, such as interleukin-1α (IL-1α) and granulocyte macrophage colony stimulatory factor (GM-CSF) in UVB-exposed epidermal keratinocytes, ubiquinol_10_, RF and FMN predominantly interrupted the IL-1α and GM-CSF-stimulated expression of NEP in dermal fibroblasts. On the other hand, as for the UVA-associated mechanism, similar to the abrogating effects reported for AX and FMN**,** ubiquinol_10_ but not RF had the potential to abrogate the increased expression of NEP and matrix-metalloproteinase-1 in UVA-exposed human fibroblasts. Our findings strongly support the *in vivo* anti-wrinkling effects of ubiquinol_10_ and AX on human and animal skin and provide convincing proof of the UV-induced wrinkling mechanism that essentially focuses on the over-expression of NEP by dermal fibroblasts as an intrinsic causative factor.

## Introduction

Ultraviolet irradiation serves as a harmful stimulus for human body and local homeostasis via the recently unveiled skin stress response mechanisms [1,2]. Repetitive ultraviolet irradiation is the most plausible factor that causes skin wrinkle which is a specific symptom for deranged skin homeostasis. In fact, repetitive exposure of the skin to UVB or UVA radiation at suberythemal doses significantly reduces its elastic properties, which in turn leads to the formation of wrinkles in animal skin [3]. In human clinical studies, the formation of facial wrinkles has been predominantly attributed to the marked loss of elastic properties in the affected dermis [4,5,6]. Repetitive UVB or UVA exposure of animal skin also elicits a marked alteration in the three-dimensional structure of elastic fibers in the exposed dermis, which is closely linked to the subsequent reduction in skin elastic properties [7,8]. The three-dimensional alteration of elastic fibers has been substantiated by the marked and continuous up-regulation of an elastin-degrading enzyme in wrinkled skin after repetitive UVB or UVA irradiation [9,10] as well as after ovariectomy [11]. The sum of those studies strongly indicates that the up-regulated activity of skin fibroblast-derived elastase plays a pivotal role in the wrinkling of skin via the impairment of elastic fiber configuration and the subsequent loss of skin elasticity. Although the skin fibroblast-derived elastase had never been clarified genetically, we recently identified it as a previously known enzyme, neprilysin, also known as neutral endopeptidase (NEP) [12]. This discovery enabled us to determine the expression of this enzyme at the transcriptional and translational levels in human fibroblasts. Therefore, we recently characterized the epithelial-mesenchymal paracrine cytokine interactions between UVB-exposed-keratinocytes and dermal fibroblasts in a co-culture (human fibroblasts) system with cell culture inserts (human keratinocytes) or by incubating the conditioned medium from UVB-exposed-keratinocytes with human fibroblasts. Both of those models identified the up-regulation of NEP expression in human fibroblasts [13, 14, 15] and we found that interleukin-1α (IL-1α) and granulocyte macrophage colony stimulatory factor (GM-CSF) are intrinsic cytokines secreted by UVB-exposed keratinocytes that specifically stimulate the expression of NEP by fibroblasts [13, 14, 15]. On the other hand, because of the deeper penetration of UVA into the dermis where fibroblasts are located, compared with UVB radiation which has a limited permeability to the epidermis only, UVA-induced photo-damage is believed to be attributable to the directly induced cellular alteration(s) of dermal fibroblasts. We recently found that the direct UVA exposure of human fibroblasts also elicits a significant increase in the expression of matrix metallo-protease (MMP)-1 as well as NEP at the transcriptional, translational and enzymatic levels [16, 17]. This suggested that UVA-induced wrinkle formation is mediated at least via the up-regulated activity of NEP and/or MMP-1 derived from dermal fibroblasts. The sum of this evidence strongly indicates the possibility that substances with anti-wrinkling properties could preferentially have inhibitory effects on both the UVB-associated epithelial-mesenchymal cytokine interaction- and the UVA-induced up-regulation of NEP in fibroblasts.

It is well established that UV irradiation rapidly generates reactive oxygen species (ROS) in the exposed cells or tissues [18, 19], the excessive formation of which is thought to elicit various types of photo-damage in the skin including wrinkles via stress-activated intracellular signaling cascades. Consistently, anti-oxidants such as ubiquinol_10_ [20, 21] and astaxanthin (AX) [22, 23] have been documented to have anti-wrinkling potential in animal and in human skin. Although it is easily assumed that their ROS depleting effects mainly contribute to their anti-wrinkling mechanisms, we recently discovered that several abrogating effects of anti-oxidants on UV-induced biological events cannot be necessarily accounted for by the depletion of generated ROS. This is based upon the fact that such biological effects can be elicited even if the anti-oxidants are treated post-irradiation even though the lifetime of ROS is too short (e.g. the lifetime of •O2 is 4 μs) [24] to be sufficient to deplete the generated ROS when treated immediately after UV radiation. These ROS depletion-independent mechanisms occur in the abrogating effects elicited by AX on the UVB-induced secretion of prostaglandin E_2_ and inflammatory cytokines such as IL-1α and IL-8 as well as on the protein expression of cyclooxygenase (COX)-2 [25] and transglutaminase I [26] in UVB-exposed human keratinocytes, and in the abolishing effect of French maritime pine bark extract on the increased expression of endothelin B receptor (EDNRB) and tyrosinase in UVB-exposed human melanocytes [27]. A similar ROS depletion-independent mechanism also occurs in the suppressive effect of AX on the stem cell factor (SCF)-stimulated pigmentation in non-UV-exposed human epidermal equivalents [28] as well as in the abrogating effects of post-irradiation treatment with AX on the UVA-stimulated expression of NEP in human fibroblasts [16]. Such a novel anti-oxidant mechanism prompted us to determine the mode of action of various anti-oxidants, including ubiquinol_10_ and AX by post-irradiation treatment in association with their possible anti-wrinkling mechanism through which the UV-induced up-regulation of wrinkle-inducing enzymes (NEP and MMP-1) is attenuated in human fibroblasts. The UV light included in sunlight consists of UVB and UVA, both of which have different mechanisms of action even though both are involved in the up-regulated levels of NEP. We have already reported the abrogating effects of flavin mononucleotide (FMN) and AX on the UVA-induced up-regulation of NEP and MMP-1 in human fibroblasts [16, 17]. Therefore, in this study, to characterize the possible anti-wrinkling effects elicited by post-irradiation treatment with 4 anti-oxidants, ubiquinol_10_, riboflavin (RF), FMN and AX, we compared the interrupting effects of those anti-oxidants on UVB-associated epithelial-mesenchymal cytokine interactions leading to the up-regulation of NEP in human fibroblasts. We then determined the abrogating effects of ubiquinol_10_ and RF on the up-regulation of NEP and MMP-1 in UVA-exposed human fibroblasts in comparison with published studies on the similar effects of AX and FMN.

In this study, we show that all 4 anti-oxidants tested have an interrupting effect on the UVB-associated epithelial-mesenchymal cytokine interactions that lead to the up-regulation of NEP in human fibroblasts but with different modes of action. While AX mainly serves as an inhibitor for the secretion of wrinkle-inducing cytokines, such as IL-1α and GM-CSF, in UVB-exposed epidermal keratinocytes, ubiquinol_10_, RF and FMN interrupt the IL-1α and GM-CSF-stimulated expression of NEP in dermal fibroblasts. On the other hand, as for UVA-associated mechanisms, similar to the abrogating effects observed with AX [16] and FMN [17], ubiquinol_10_ but not RF has the potential to abrogate the increased expression of NEP and MMP-1 in UVA-exposed human fibroblasts.

## Materials and Methods

### Materials

The synthetic substrate for elastase, N-succinyl-tri-alanyl-p-nitroaniline (STANA) was purchased from the Peptide Institute Inc (Osaka, Japan). The anti-human NEP (neutral endopeptidase) antibody was purchased from Santa Cruz Biotechnology (CA, USA). Rabbit anti-human MMP-1 (Collagenase Type1) rabbit antibody was purchased from Sigma (MO, USA). Horseradish peroxidase conjugated goat polyclonal anti-mouse IgG was obtained from Transduction Laboratories (NJ, USA), ELISA kits (for IL-1α, IL-1ß, IL-6, IL-8, GM-CSF and endothelin (EDN)-1) were obtained from Endogen (Thermo Fisher Scientific, Yokohama, Japan). Ubiquinol_10_ was kindly provided by Kaneka (Kaneka QH^™^ from Kaneka) via Dr. YY. Human primary keratinocytes (HPKs), human dermal fibroblasts (HDFs), serum-free keratinocyte growth medium (Medium 154S) containing low calcium (0.2 mM), bovine pituitary extract (BPE) and epidermal growth factor (EGF), were obtained from Kurabo (Tokyo, Japan). HaCaT human keratinocytes were kindly supplied by Dr. M. Furue (Kyushu University, School of Medicine, Department of Dermatology). Recombinant human GM-CSF (215-GM-010) was purchased from R&D Systems (MN, USA). Recombinant human IL-1α (200-01A) was obtained from PEPROTECH (London, UK). Other chemicals of reagent grade were purchased from Sigma-Aldrich (MO, USA).

### Cell cultures

HDFs derived from human foreskins (Cell Applications, Inc., CA, USA) were cultivated in Dulbecco’s modified Eagle’s medium (DMEM) with 10% fetal bovine serum (FCS)/100 μg/ml penicillin, 100μg/ml streptomycin and 250 ng/ml amphotericin B at 37°C in a 95% air, 5% CO_2_ atmosphere. HPKs were maintained in serum-free keratinocyte medium (Medium 154S) (Kurabo, Tokyo, Japan) supplemented with 5 ng/ml EGF and 50 μg/ml BPE at 37°C with 5% CO_2_. HaCaT cells were maintained in DMEM with 10% FCS, 100 μg/ml penicillin, 100μg/ml streptomycin and 250 ng/ml amphotericin B at 37°C in a 95% air, 5% CO_2_ atmosphere. In experiments to measure cytokine levels using ELISA kits, HPKs, HaCaT cells and HDFs were cultured in DMEM without FCS after UVB or UVA irradiation.

### Co-cultures with cell culture inserts

Co-cultures with cell culture inserts were performed as previously detailed [13]. HPKs or HaCaT cells were seeded in Medium 154S supplemented with 5 ng/ml EGF and 50 μg/ml BPE or in DMEM with 10% FCS, respectively, at 37°C with 5% CO_2_ at a density of 5 x 10^6^ cells/well in 6 well format cell culture inserts with translucent membranes and 1.0μm pores (Becton Dickinson, NJ, USA). The cell culture inserts were then inverted and placed in Multiwell^™^ 6 well plates (Becton Dickinson) where HDFs had been seeded in DMEM with 10% FCS, 100 μg/ml penicillin, 100 μg/ml streptomycin and 250 ng/ml amphotericin B at 37°C in a 95% air, 5% CO_2_ atmosphere at a density of 5 x 10^6^ cells/well. After the co-culture units with the cell culture inserts had been maintained for 12 h in Medium 154S without EGF and BPE (for HPKs) or in DMEM without FCS (for HaCaT cells), the cell culture inserts were exposed to UVB after exchanging the Medium 154S or DMEM with phosphate-buffered saline (PBS). After UVB irradiation, the cell culture inserts were again inverted and placed in 6 well plates and the co-culture units were cultured in Medium 154S without EGF and BPE (for HPKs) or in DMEM without FCS (for HaCaT cells) for 48–72 h as noted.

### Extraction of NEP

Extraction of NEP was performed according to the method previously detailed [13, 16]. Cultured HDFs were washed once with PBS, then were scraped into PBS and centrifuged at 4°C, 1000 rpm for 5 min. The cell pellets were lysed with 0.1 M Tris–HCl (pH 7.6) buffer containing 0.1% Triton-X 100 and 1 mM phenylmethylsulfonylfluoride (PMSF), followed by ultrasonication for 5 min on ice. Cleared supernatants after the removal of cell residues by centrifugation (2000 rpm, 10 min) were used as the fibroblast enzyme solution.

### UVB irradiation

UVB exposure of HPKs or HaCaT cells was performed as previously detailed [25, 26, 29]. Briefly, cells cultured in 10 cm dishes or in cell culture inserts were washed twice with PBS and were then exposed to UVB. The UVB source was a SE fluorescent lamp (Clinical Supply, Tokyo, Japan) that emitted an energy spectrum with high fluency in the UVB region (280–320 nm) with a peak at 305 nm. The emitted dose was calculated using a UVB radiometer photodetector (Torex, Tokyo, Japan). The duration of UVB irradiation delivered to cells was altered by sliding a plastic lid covered with aluminum foil onto the flat-bottomed plate. After UVB irradiation, the cells or the co-culture units were maintained for 48–72 h in DMEM without FCS (for HaCaT cells) or in Medium 154S without EGF and BPE (for HPKs) at 37°C in a 95% air, 5% CO_2_ atmosphere. Control samples were mock-irradiated and were maintained under the same culture conditions as those used for the UVB-irradiated specimens.

### UVA Irradiation

HDFs were irradiated with UVA as previously detailed [16]. The UVA irradiation source was a FL20S/BLB fluorescent lamp (Clinical Supply, Tokyo, Japan) that emitted an energy spectrum with high fluency in the UVA region (300–430 nm), with a peak at 352 nm. A 6-mm thick glass plate was used to block UVB emissions. The emitted dose was calculated using a UVA radiometer photodetector (Torex, Tokyo, Japan). The cells were washed with PBS, then were immersed in PBS and subjected to UVA irradiation. The duration of UV irradiation delivered to cells was altered by sliding a plastic lid covered with aluminum foil onto a flat-bottomed plate. After irradiation, the cells were cultured in DMEM without FCS at 37°C. Control samples were mock-irradiated and maintained under the same culture conditions as those used for the UVA-irradiated specimens.

### Cytokine measurements

HPKs or HDFs were cultured at a density of 1.0 x 10^5^ cells/well in 6 well format cell culture inserts, which were placed in 6 well plates or in 6 or 10 cm dishes, respectively, and were exposed to UVB at the indicated doses. The secretion levels of IL-1α, IL-1ß, IL-6, IL-8, GM-CSF, tumor necrosis factor α (TNFα) and endothelin (EDN)-1 in the media were then measured using ELISA kits at the indicated time of post-irradiation. Secretion levels are expressed as pg/ml.

### MMP-1 activity assay

For MMP-1 activity, analyses were performed as previously detailed [16]. Briefly, fibroblast culture media was concentrated with a Centricon at 4°C by approximately 10-fold and 80 μl of the concentrate was incubated for 15 min at 37°C with trypsin solution (0.05 mg/ml) to activate MMP-1. After the addition of 10 μl trypsin inhibitor solution (0.25 mg/ml) to inactivate the trypsin, the concentrated media was measured for MMP-1 activity using a type I collagenase assay kit (Code No. AK37; Primary Cell Co., LTD) which measures the MMP-1-mediated cleavage of fluorescently-labeled collagen type I, according to the manufacturer’s instructions.

### Measurement of elastase activity

Elastase activity using the synthetic substrate STANA was measured as previously detailed [12, 13, 16]. Briefly, 100 μl enzyme solution was dispensed into 96-well plates, which were pre-incubated for 15 min at 37°C. After addition of 2 μl 62.5 mM STANA, further incubation was performed for 1 h at 37°C. The release of p-nitroaniline (p-NA) was measured by absorbance at 405 nm and enzymatic activity is expressed as unit per μg protein, one unit representing the activity that releases 1 nmol of p-NA per h.

### Western blotting

For NEP determination, western blotting was performed as previously detailed [12, 13, 16]. Briefly, cell lysates were subjected to 10% SDS-PAGE and blotted to PVDF membranes (Bio-Rad, CA, USA). After blocking with 3% bovine serum albumin-containing Tris/HCl (pH 7.5) with 100 mM NaCl, the membranes were treated with the mouse monoclonal anti-human NEP antibody at room temperature for 1 h, then with anti-mouse IgG conjugated to horseradish peroxidase. They were then treated with Amersham ECL reagents and exposed to x-ray film for specified times to detect bands. For MMP-1 determinations, as described previously [11, 14], fibroblast medium was concentrated in CentriconTM centrifugal filter devices (Mr cutoff 30,000) for 35 min at 5,000 x g at 4°C. Volume equivalents were assayed for MMP-1 by SDS-PAGE and immunoblotting with the anti-MMP-1 antibody. In each case, immunoblots were resolved by incubation with a horseradish peroxidase-conjugated anti-rabbit antibody, then were treated with Amersham ECL reagents and exposed to x-ray film for the specified times to detect bands. The specific bands were quantitated by densitometric scanning and were analyzed with Quantity One^®^ (Bio Rad).

### Real-time RT-PCR analysis

Real-time RT-PCR analyses were performed as previously detailed [13, 28, 30]. Briefly, after total RNAs were purified using an RNeasy Mini Kit (Qiagen, CA, USA), cDNAs were synthesized with a Rever Tra Ace qPCR RT Kit (Toyobo, Tokyo, Japan) by reverse transcription of 1μg total RNA using oligo dT and Moloney murine leukemia virus reverse transcriptase. Real-time RT-PCR was performed with SYBR Green by Power SYBR Green PCR (Applied Biosystems Japan, Tokyo, Japan) in a DNA engine Opticon Real-Time PCR detection system (MJ Japan Ltd., Tokyo, Japan). After initial denaturation at 94°C for 15 min, amplification in the DNA engine Opticon was conducted for 40 cycles at 94°C for 15 s, at 53°C for 30 s and at 74°C for 1 min. Primers used are described in Table 1.

**Table 1:** Primer sequences used in this study.

GAPDH	Forward	5’-GAAGGTGAAGGTCGGAGTCAACG-3’
	Reverse	5’-AGTCCTTCCACGATAACCAAAGTTG-3’
Neutral Endopeptidase (NEP)	Forward	5’-GAACCTACAAGGAGTCCAGA-3’
	Reverse	5’-GCTCCACTTATCCACTCATC-3’
Collagenase-I (MMP-1)	Forward	5’-GCTGGGAGCAAACACATCTGAGGT-3’
	Reverse	5’-TGAGCCGCAACACGATGTAAGTTG-3’
Elastin	Forward	5’-AGGTGTATACCCAGGTGGCGTGCT-3’
	Reverse	5’-CAACCCCTGTCCCTGTTGGGTAAC-3’
Collagen I	Forward	5’-CTGGTCCCCAAGGCTTCCAAGGTC-3’
	Reverse	5’-CCATCATTTCCACGAGCACCAGCA-3’

### DNA microarray analysis

HPKs were exposed to UVB radiation at 80 mJ/cm^2^ and were cultured for up to 72 h. At several times after UVB radiation, total RNAs were isolated using the Trizol reagent (Invitrogen) according to the manufacturer’s instructions. DNA microarray analysis was performed using GenopalTM Allergy Chips (Mitsubishi Rayon, Tokyo, Japan) according to the manufacturer’s protocol.

### Statistical analysis

All data are expressed as means ± SD (n = 3) unless noted otherwise. For pairwise comparisons, either Student’s t-test or Welch’s t-test was applied. For multiple comparisons, data were tested by one-way ANOVA, and subsequently using the Tukey or Dunnet multiple comparison test. P values less than 0.05 are considered statistically significant.

## Results

### Effects of UVB/UVA exposure of human keratinocytes or human fibroblasts on the expression of NEP at the protein and enzymatic levels in human fibroblasts

**Fig 1:**
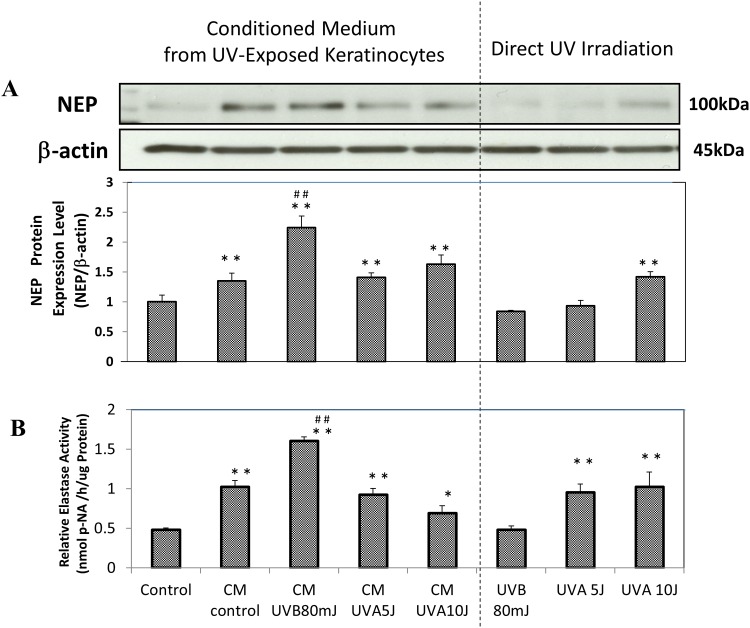
Effects of UVB/UVA exposure of human keratinocytes or human fibroblasts on the expression of NEP at the protein and enzymatic levels in human fibroblasts. **CM control:** HDFs treated for 72 h with conditioned medium from non-irradiated HaCaT cells, **CM/UVB80mJ:** HDFs treated for 72 h with conditioned medium from UVB (80 mJ/cm^2^)-irradiated HaCaT cells, **CM/UVA5J:** HDFs treated for 72 h with conditioned medium from UVA (5 J/cm^2^)-irradiated HaCaT cells, **CM/UVA10J:** HDFs treated for 72 h with conditioned medium from UVA (10 J/cm^2^)-irradiated HaCaT cells, **UVB/80mJ:** UVB (80 mJ/cm^2^)-irradiated HDFs at 72 h post-irradiation, **UVA5J:** UVA (5 J/cm^2^)-irradiated human fibroblasts at 72 h post-irradiation**, UVA10J:** UVA (10 J/cm^2^)-irradiated HDFs at 72 h post-irradiation**,** n = 3**,** *: p<0.05, **: p<0.01 vs control, ##: p<0.01 vs CM control. **p-NA**: p-nitroaniline.

When the conditioned medium from UVB- or UVA-exposed HaCaT cells was incubated for 72 h with HDFs in culture, the protein expression and enzymatic activity of NEP was significantly increased by the UVB (80 mJ/cm^2^)-conditioned medium, but not by the UVA (5 and 10 J/cm^2^)-conditioned medium, compared with non-exposed conditioned medium (Fig 1). On the other hand, when HDFs were directly exposed to UVB or UVA radiation, the expression of NEP at 72 h post-irradiation was significantly increased by UVA (10 J/cm^2^) at both the protein and enzymatic levels and by UVA (5 J/cm^2^) at the enzymatic level, but not by UVB (80 mJ/cm^2^) at either the protein or enzymatic level and by UVA (5 J/cm^2^) at the protein level compared with the non-exposed control (Fig 1).

### GM-CSF secretion in UVB/UVA-exposed human keratinocytes and the effects of the conditioned medium on GM-CSF secretion by HDFs

**Fig 2:**
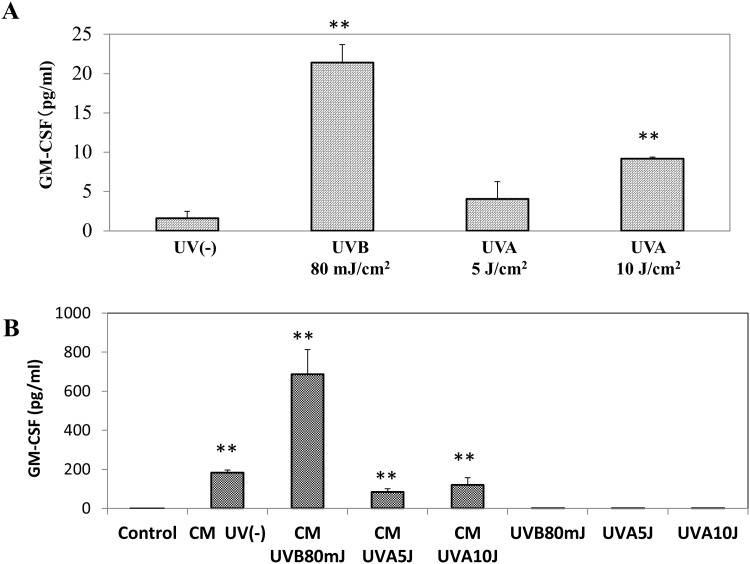
Effects of UVB/UVA exposure of human keratinocytes or HDFs on the secretion of GM-CSF by HDFs. **A:** HaCaT cells**, UV(-):** Conditioned medium from non-irradiated HaCaT cells**, UVB80mJ:** Conditioned medium from UVB (80 mJ/cm^2^)-irradiated HaCaT cells at 72 h post-irradiation**, UVA5J:** Conditioned medium from UVA (5 J/cm^2^)-irradiated HaCaT cells at 72 h post-irradiation, **UVA10J:** Conditioned medium from UVA (10 J/cm^2^)-irradiated HaCaT cells at 72 h post-irradiation**,** n = 3, **: p<0.01 vs CM UV(-); **B:** HDFs, **CM UV(-):** Conditioned medium of HDFs treated for 72 h with conditioned medium from non-irradiated HaCaT cells at 72 h sham-irradiation**, CM/UVB80mJ:** Conditioned medium of HDFs treated for 72 h with conditioned medium from UVB (80 mJ/cm^2^)-irradiated HaCaT cells at 72 h post-irradiation**. CM/UVA5J:** Conditioned medium of HDFs treated for 72 h with conditioned medium from UVA (5 J/cm^2^)-irradiated HaCaT cells at 72 h post-irradiation, **CM/UVA10J:** Conditioned medium of HDFs treated for 72 h with conditioned medium from UVA (10 J/cm^2^)-irradiated HaCaT cells at 72 h post-irradiation, **UVB80mJ:** Conditioned medium from UVB (80 mJ/cm^2^)-irradiated HDFs at 72 h post-irradiation**, UVA5J:** Conditioned medium from UVA (5 J/cm^2^)-irradiated HDFs at 72 h post-irradiation, **UVA10J:** Conditioned medium from UVA (10 J/cm^2^)-irradiated HDFs at 72 h post-irradiation, n = 3, **: p<0.01 vs control.

When HaCaT cells or HDFs were exposed to UVB/UVA radiation, the secretion of GM-CSF was significantly increased at 72 h post-irradiation in the medium of UVB- (80 mJ/cm^2^) or UVA- (10 J/cm^2^) exposed HaCaT cells, but not in the medium of UVA- (5 J/cm^2^) exposed HaCaT cells (Fig 2). In contrast, the secretion level of GM-CSF did not change at 72 h post-irradiation in the medium of UVB- (80 mJ/cm^2^) or UVA- (5 and10 J/cm^2^) exposed HDFs. On the other hand, when those conditioned media were added and incubated for 72 h with HDFs in culture, the secretion of GM-CSF by HDFs was markedly increased by the conditioned medium from non-, from UVB- (80 mJ/cm^2^) and from UVA- (5 and10 J/cm^2^) exposed cells compared to the control without the conditioned medium, in which only the conditioned medium from UVB-exposed cells significantly stimulated to the greatest extent the GM-CSF secretion compared to that from non-exposed cells (Fig 2).

### Abrogation by anti-oxidants of the stimulated NEP activity elicited by UVB-exposed human keratinocytes in the co-culture system with cell culture inserts

**Fig 3:**
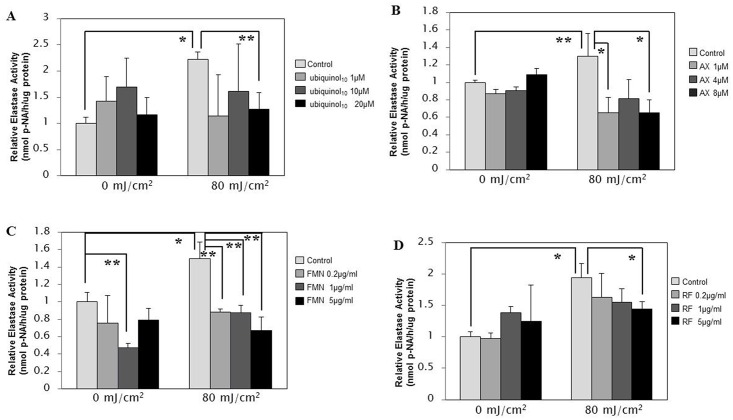
Abrogation by anti-oxidants of the stimulated NEP activity elicited by UVB-exposed human keratinocytes in the co-culture system. The co-culture units consisting of UVB- or non-exposed HaCaT cells (in the cell inserts) and HDFs (in the well plates) were treated for 48 h with each of the 4 anti-oxidants at the indicated concentrations immediately after UVB irradiation and the elastase activity in HDFs co-cultured in the well plates was then measured as described in the **Materials and Methods**. **A**: treated with ubiquinol_10_; **B**: treated with AX; **C**: treated with FMN; **D**: treated with RF. **AX**: Astaxanthin, **FMN**: Flavin Mononucleotide, **RF**: Riboflavin, **p-NA**: p-nitroaniline, n = 3, **: p<0.01, *: p<0.05.

Since HDFs co-cultured with UVB-exposed human keratinocytes significantly increased the expression of NEP at the transcriptional, translational and enzymatic levels, a mode of action that imitates the UVB-induced wrinkling mechanism [13], we examined the inhibitory effects of the four anti-oxidants on the stimulated activity of the elastase to characterize their possible anti-wrinkling potential. Thus, when co-culture units consisting of UVB- or non-exposed HaCaT cells (in the cell inserts) and HDFs (in the well plates) were cultured for 48 h in the presence or absence of these anti-oxidants at the indicated concentrations immediately after UVB irradiation, the elastase activity in HDFs co-cultured in the well plates was significantly up-regulated at 48 h post-irradiation, the stimulation of which was markedly abrogated by the addition of each of the 4 anti-oxidants tested (Fig 3A/3B/3C/3D).

### Time course of gene expression for several inflammation-associated factors including IL-1α and GM-CSF in UVB-exposed human keratinocytes

**Fig 4:**
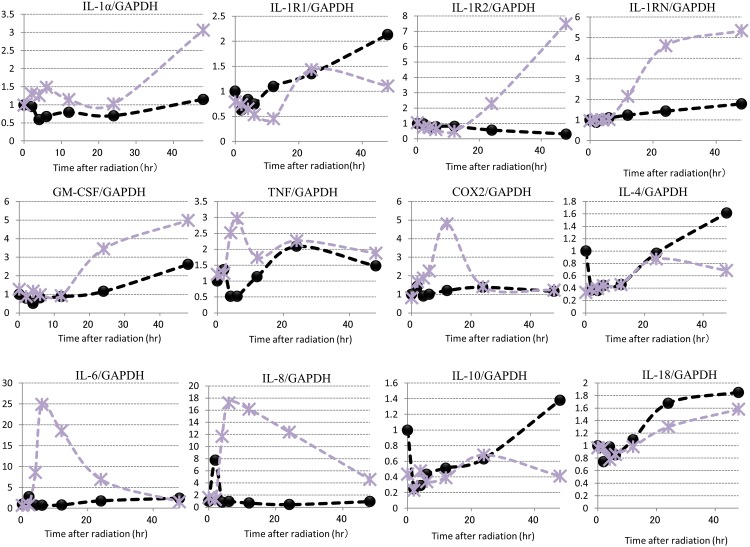
Time course of gene expression levels of cytokines and receptors including IL-1α and GM-CSF in UVB-exposed HPKs. *--●--*:*UVB -*, --*--: *UVB 80 mJ/cm*^*2*^. HPKs in culture were exposed to UVB at a dose of 80 mJ/cm^2^ and were subjected to DNA microarray analysis at the indicated post-irradiation times. **IL:** Interleukin, **IL-1R1**: Interleukin-1 receptor-1; **IL-1R2**: Interleukin-1 receptor-2; **IL-1RN**: Natural interleukin-1 receptor antagonist, **GM-CSF:** Granulocyte macrophage colony stimulatory factor, **TNFα:** Tumor necrosis factor, **COX2:** Cyclooxygenase-2, **IL-4:** Interleukin-4, **IL-6:** Interleukin-6, **IL-8:** Interleukin-8, **IL-10:** Interleukin-10, **IL-18:** Interleukin-18.

To elucidate the autocrine mechanism(s) involved in the secretion of NEP-inducing cytokines [13] in UVB-exposed human keratinocytes, we used DNA microarray analysis to examine the time course of the gene expression patterns of several inflammation-associated factors including IL-1α and GM-CSF, IL-1α receptor, natural IL-1 antagonist (IL-1RN) and cyclooxygenase (COX)-2 in UVB-exposed HPKs (Fig 4). Comparison of the time course of the expression of all genes tested revealed that the mRNA levels for IL-1α, TNFα, COX-2, IL-6 and IL-8 were significantly increased at 3–5 h post-irradiation in UVB-exposed HPKs, whereas mRNA levels for IL-1R2, IL-1RN and GM-CSF were accentuated at ~20 h post-irradiation. In contrast, the mRNA levels for IL-4, IL-10 and IL-18 were significantly down-regulated at 20 ~ 40 h post-irradiation.

### The secretion of IL-1α and GM-CSF in UVB-exposed HPKs

**Fig 5:**
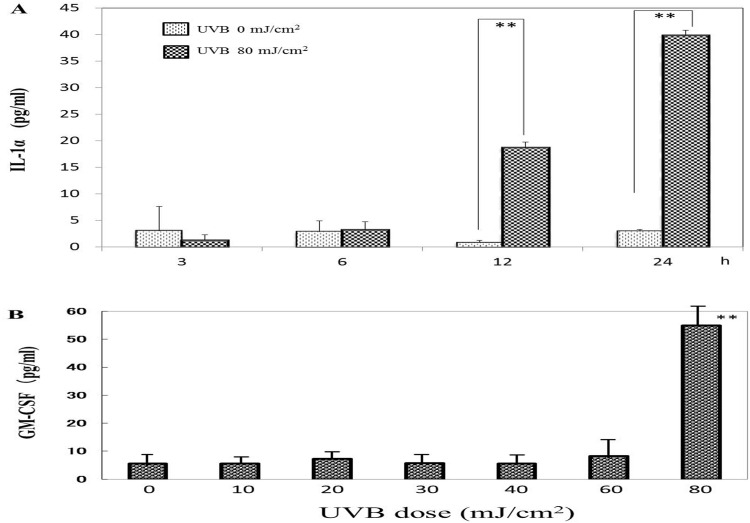
The secretion of IL-1α and GM-CSF in UVB-exposed HPKs. ***A*:** IL-1α secretion; After HPKs in culture were exposed to UVB at a dose of 80 mJ/cm^2^, the secretion levels of IL-1α were measured by ELISA at the indicated times post-irradiation with or without UVB. ***B***: GM-CSF secretion; After HPKs in culture were exposed to UVB at the doses noted, the secretion levels of GM-CSF were measured by ELISA at 48 h post-irradiation. n = 3, **: p<0.01 vs non-UVB irradiation.

When the levels of wrinkling-inducing cytokines, IL-1α and GM-CSF, were measured by ELISA in UVB-exposed HPKs, the secretion levels of IL-1α were significantly increased at 12 h post-irradiation and were further accentuated at 24 h post-irradiation (Fig 5A). The increased secretion of GM-CSF was detected at 48 h post-irradiation only following UVB irradiation at a dose of 80 mJ/cm^2^ (Fig 5B). We have already reported that the secretion level of GM-CSF in UVB (80 mJ/cm^2^)-exposed HPKs increases at 20–24 h post-irradiation in the same conditions [13].

### Effects of anti-oxidants on the UVB-induced secretion of wrinkle-inducing inflammatory cytokines

**Fig 6:**
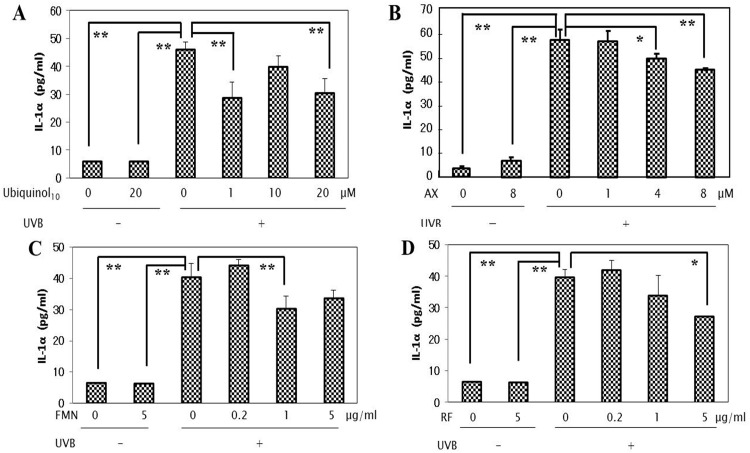
Effects of anti-oxidants on the UVB-induced secretion of the inflammatory cytokine interleukin (IL)-1α. Each of the 4 anti-oxidants were added at the indicated concentrations to HaCaT cells in culture immediately after UVB irradiation at a dose of 80 mJ/cm^2^ and after 72 h, the secretion levels of IL-1αwere measured by ELISA. **A**: treated with ubiquinol_10_; **B**: treated with AX; **C**: treated with FMN; **D**: treated with RF. **AX**: Astaxanthin, **FMN**: Flavin Mononucleotide, **RF**: Riboflavin, n = 3, **: p<0.01, *: p<0.05.

**Fig 7:**
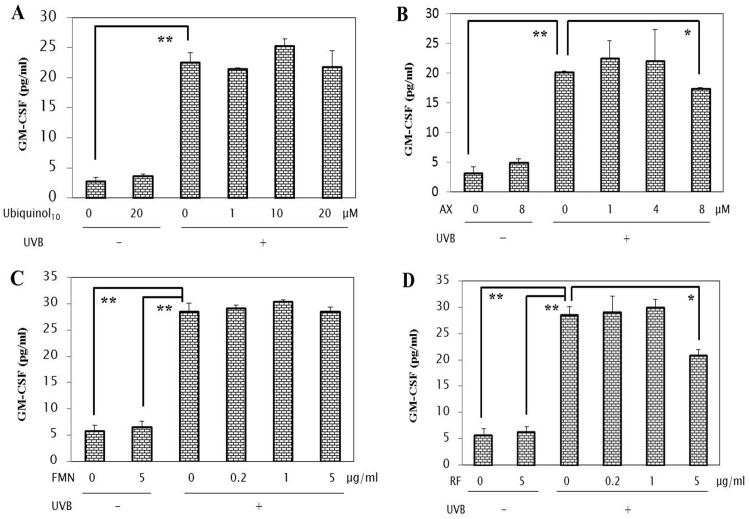
Effects of anti-oxidants on the UVB-induced secretion of the inflammatory cytokine GM-CSF. Each of the 4 anti-oxidants were added at the indicated concentrations to HaCaT cells in culture immediately after UVB irradiation at a dose of 80 mJ/cm^2^ and after 72, the secretion levels of GM-CSF were measured by ELISA. **A**: treated with ubiquinol_10_; **B**: treated with AX; **C**: treated with FMN; **D**: treated with RF. **AX**: Astaxanthin, **FMN**: Flavin Mononucleotide, **RF**: Riboflavin, n = 3, **: p<0.01, *: p<0.05.

To elucidate the mode(s) of action of the anti-oxidants tested by which the stimulated NEP activity is abrogated, we next determined whether the anti-oxidants tested have an abrogating effect on the UVB-induced secretion of the wrinkle-inducing keratinocyte-derived inflammatory cytokines, IL-1α and GM-CSF. When those anti-oxidants were added at the indicated concentrations to HaCaT cells in culture immediately after UVB irradiation, the increased secretion of IL-1α and GM-CSF at 72 h post-irradiation was significantly abrogated by the addition of each of the 4 anti-oxidants tested but with different effective concentrations (Figs 6 and 7). Thus, while ubiquinol_10_, AX, FMN and RF elicited significant suppressive effects on the increased secretion of IL-1α at concentrations of 1/20 μM, 4/8 μM, 1 μg/ml, and 5 μg/ml, respectively at 72 h post-irradiation (Fig 6), AX and RF, but not ubiquinol_10_ and FMN, significantly abrogated the increased secretion of GM-CSF at concentrations of 8 μM and 5 μg/ml, respectively (Fig 7).

### Effects of inhibiting the activation of NFkB and MSK1 on the UVB-induced secretion of GM-CSF

**Fig 8:**
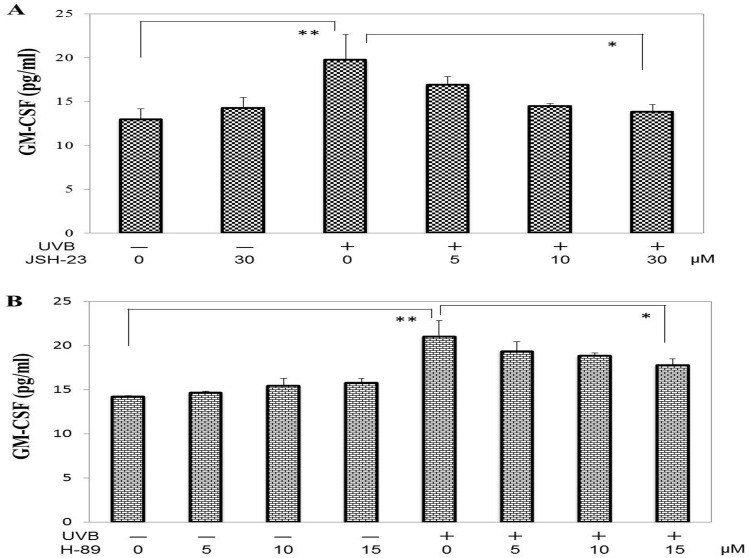
Effects of inhibiting the activation of NF-kB and MSK1 on the UVB-induced secretion of GM-CSF. **A: Effect of JSH-23,** an inhibitor for NFkB translocation inhibitor**, B: Effect of H89,** an inhibitor of MSK1. The secretion levels of GM-CSF were measured by ELISA at 72 h post-irradiation in the medium of UVB (80 mJ/cm^2^) exposed HaCaT cells after pre- or post-irradiation treatment with JSH-23 or H89, respectively, at the indicated concentrations. n = 3, **: p<0.01, *: p<0.05.

We have already reported that the increased secretion of IL-8 and prostaglandin E_2_ as well as the increased expression of the keratinization-specific enzyme transglutaminase 1 in UVB-exposed human keratinocytes is mediated at least via the p38/MSK1/NFkBSer276 axis within the NFkB signaling pathway and that some anti-oxidants, such as AX and a French maritime pine bark extract, significantly abrogate the UVB-induced up-regulation of those inflammatory and keratinization factors as well as melanocyte-specific proteins such as EDNRB and tyrosinase in human keratinocytes [25, 26] and in human melanocytes [27], respectively, by a post-irradiation treatment similar to this study. Thus, to associate the inhibitory effects of the anti-oxidants on the secretion of GM-CSF with the interruption of UVB-activated signaling cascades, we determined the effect of inhibitors for intercellular signaling of NFkB and MSK1 on the increased secretion of GM-CSF in UVB-exposed human HaCaT keratinocytes. We found that the UVB-increased secretion of GM-CSF was significantly abrogated by pre- or post-irradiation treatment with JSH-23 or H89, respectively, at 72 h post-irradiation (Fig 8). This indicates that the UVB-up-regulated secretion of GM-CSF is mediated at least via the p38/MSK1/NFkBSer276 axis within the NFkB signaling pathway and that some anti-oxidants, including the 4 anti-oxidants tested in this study, may interrupt this signaling cascade, resulting in the abrogation of the UVB-induced increase in GM-CSF secretion.

### Effects of anti-oxidants on cytokine-stimulated NEP activity

**Fig 9:**
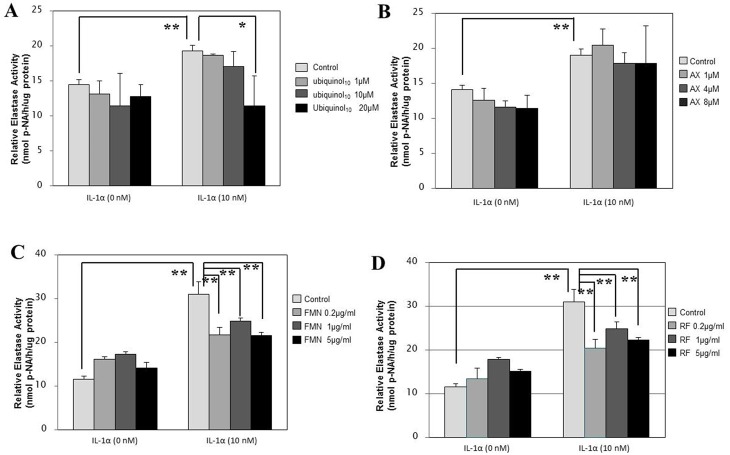
Effects of anti-oxidants on IL-1α-stimulated NEP activity in HDFs. HDFs were incubated for 72 h with IL-1α at 10 nM in the presence or absence of the various anti-oxidants at the indicated concentrations and the lysates were then subjected to the assay for the elastase activity. **A**: treated with ubiquinol_10_; **B**: treated with AX; **C**: treated with FMN; **D**: treated with RF. **AX**: Astaxanthin, **FMN**: Flavin Mononucleotide, **RF**: Riboflavin, **p-NA**: p-nitroaniline, n = 3, **: p<0.01, *: p<0.05.

**Fig 10:**
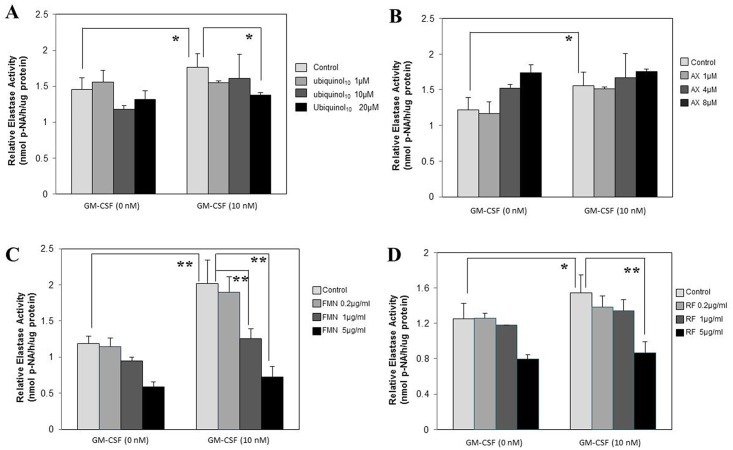
Effects of anti-oxidants on GM-CSF-stimulated elastase activity in HDFs. HDFs were incubated for 72 h with GM-CSF at 10 nM in the presence or absence of the various anti-oxidants at the indicated concentrations and the lysates were then subjected to the assay for the elastase activity. **A**: treated with ubiquinol_10_; **B**: treated with AX; **C**: treated with FMN; **D**: treated with RF. **AX**: Astaxanthin, **FMN**: Flavin Mononucleotide, **RF**: Riboflavin, **p-NA**: p-nitroaniline, n = 3, **: p<0.01, *: p<0.05.

To elucidate the mode of action of the anti-oxidants tested by which the stimulated elastase activity is abrogated in the co-culture system, we next determined whether they abrogate the IL-1α or GM-CSF-stimulated elastase activity in human fibroblasts. When HDFs were incubated for 72 h with IL-1α or GM-CSF at 10 nM in the presence or absence of those anti-oxidants, ubiquinol_10_, FMN and RF, but not AX, exhibited a significant abrogating effect on the IL-1α-stimulated elastase activity at concentrations of 20 μM, 0.2/1/5 μg/ml and 0.2/1/5 μg/ml, respectively (Fig 9). Similarly, ubiquinol_10_, FMN and RF, but not AX, elicited a significant abrogating effect on the GM-CSF-stimulated elastase activity at concentrations of 20 μM, 1/5 μg/ml and 5 μg/ml, respectively (Fig 10).

### Effects of ubiquinol_10_ and RF on the expression of collagen I mRNA in UVA-exposed HDFs

**Fig 11:**
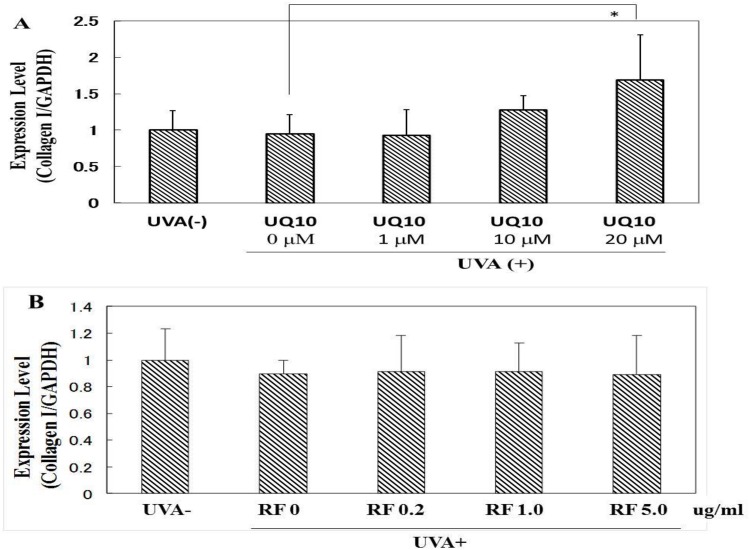
Effects of ubiquinol_10_ and RF on the expression level of collagen I mRNA. ***A*: UQ10:** ubiquinol_10,_ **B: RF.** The level of collagen I mRNA in HDFs was assessed by real-time RT-PCR at 12 h post-irradiation. **UQ10:** ubiquinol_10_, **RF**: riboflavin, n = 6, *: p < 0.05.

When HDFs were exposed to UVA irradiation at 10 J/cm^2^, although there was no change in the expression level of collagen I mRNA at 12 h post-irradiation, the addition of ubiquinol_10_ at a concentration of 20 μM significantly stimulated the expression level of collagen I mRNA (Fig 11A). However, the addition of RF at concentrations of 0.2, 1 or 5 μg/ml elicited no change in the expression of collagen I mRNA (Fig 11B).

### Effects of ubiquinol_10_ and RF on the expression level of elastin mRNA

**Fig 12:**
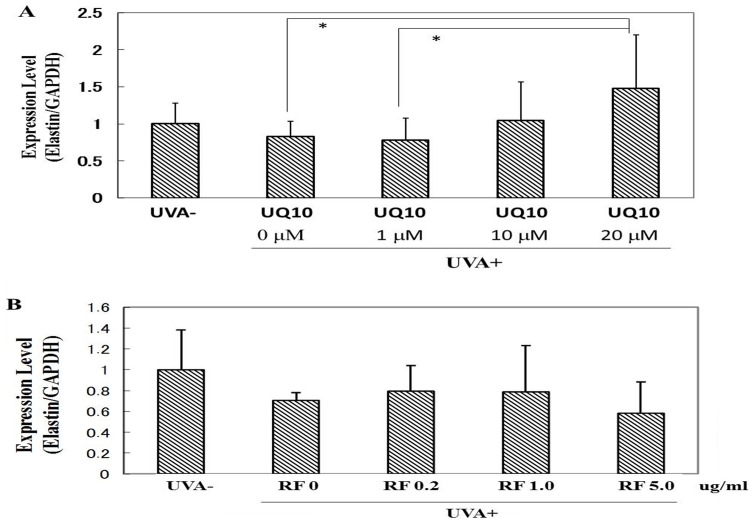
Effects of ubiquinol_10_ and RF on the expression level of elastin mRNA. **A:** ubiquinol_10_ **B:** RF. The expression level of elastin mRNA in HDFs was assessed by real-time RT-PCR at 12 h post-irradiation. **UQ10:** ubiquinol_10_, **RF**: riboflavin, n = 6, *: p < 0.05.

When HDFs were exposed to UVA irradiation at 10 J/cm^2^, although there was no change in the expression level of elastin mRNA at 12 h post-irradiation, the addition of ubiquinol_10_ at a concentration of 20 μM significantly stimulated the expression level of elastin mRNA (Fig 12A). In contrast, the addition of RF at concentrations of 0.2, 1 or 5 μg/ml elicited no change in the expression level of elastin mRNA (Fig 12B).

### Effects of ubiquinol_10_ and RF on the expression level of NEP mRNA

**Fig 13:**
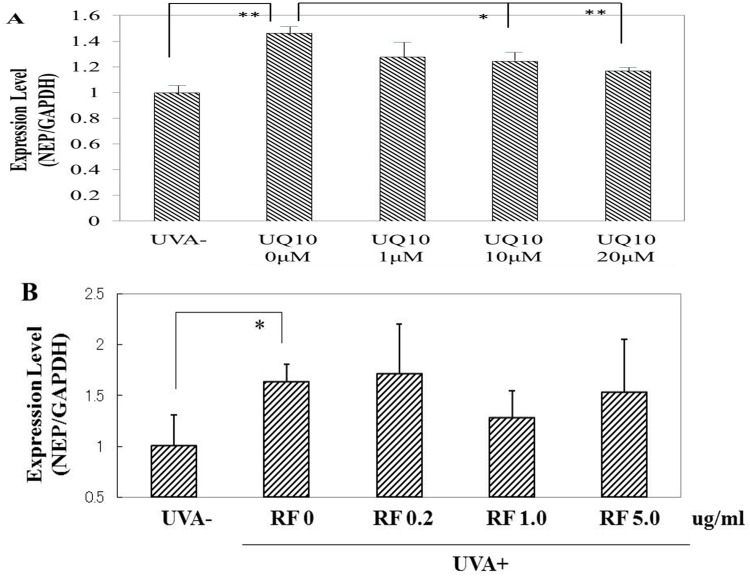
Effects of ubiquinol_10_ and RF on NEP mRNA expression in UVA-exposed HDFs. **A:** ubiquinol_10,_ **B:** RF. The mRNA level for NEP in UVA-exposed HDFs was assessed by real-time RT-PCR at 12 h post-irradiation. **UQ10:** ubiquinol_10_, **RF**: riboflavin, n = 6, *: p < 0.05, **: p<0.01.

We have already reported that when HDFs in culture are exposed to UVA at doses of 5 or 10 J/cm^2^, the expression level of NEP mRNA in HDFs was slightly but significantly increased at 12 h post-irradiation at both 5 and 10 J/cm^2^ and at 24 h post-irradiation at 5 J/cm^2^ [16]. When ubiquinol_10_ was added at concentrations of 1, 10 and 20 μM immediately after UVA irradiation at 10 J/cm^2^, the significantly increased expression level of NEP mRNA at 12 h post-irradiation remained unchanged at the concentration of 1 μM, but was significantly abrogated at concentrations of 10 and 20 μM (Fig 13A). In contrast, RF did not elicit any significant abrogating effects at any concentration tested (Fig 13B)

### Effects of ubiquinol_10_ and RF on the expression level of MMP-1 mRNA

**Fig 14:**
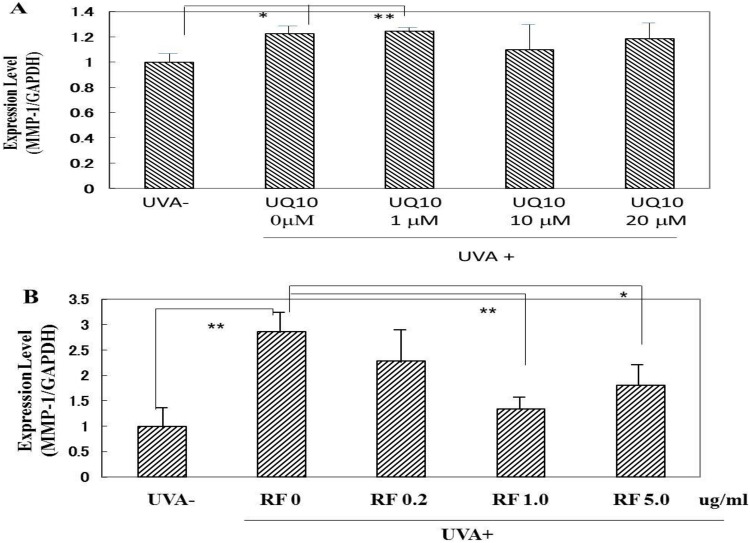
Effects of ubiquinol_10_ and RF on MMP-1 mRNA expression in UVA-exposed HDFs. **A:** ubiquinol_10,_ **B:** RF. The expression level of MMP-1 mRNA in UVA-exposed HDFs was assessed by real-time RT-PCR at 6 h post-irradiation. **UQ10:** ubiquinol_10_, **RF**: riboflavin, n = 6, *: p < 0.05, **: p<0.01.

We have already reported that when HDFs are exposed in culture to UVA at doses of 5 or 10 J/cm^2^, the expression level of MMP-1 mRNA in HDFs was significantly increased at 6, 9 and 12 h post-irradiation at both 5 and 10 J/cm^2^ and at 24 h post-irradiation at 10 J/cm^2^ [16]. When ubiquinol_10_ was added at concentrations of 1, 10 and 20 μM immediately after UVA irradiation at 10 J/cm^2^, the significantly increased expression level of MMP-1 mRNA was not changed at 12 h post-irradiation at any concentrations tested (Fig 14A). In contrast, RF elicited a significant abrogating effect on the significantly increased gene expression of MMP-1 at concentrations of 1 and 5 μg/ml (Fig 14B)

### Effects of ubiquinol_10_ and RF on the expression level of NEP protein

**Fig 15:**
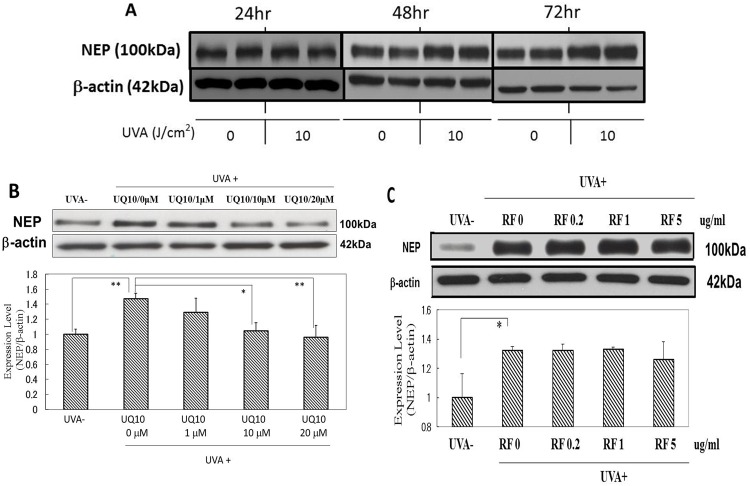
Effects of ubiquinol_10_ and RF on NEP protein levels in UVA-exposed HDFs. **A:** Time course of the expression of NEP protein after UVA exposure, **B:** treated with ubiquinol_10,_ **C:** treated with RF. The levels of NEP protein in UVA-exposed HDFs were assessed by Western blotting analysis at 72 h post-irradiation. **UQ10:** ubiquinol_10_, **RF**: riboflavin, n = 3, *: p < 0.05, **: p<0.01.

When HDFs were exposed to UVA radiation at a dose of 10 J/cm^2^, the expression level of NEP protein remained unchanged at 24 h post-irradiation, but was distinctly increased at 48 and 72 h post-irradiation (Fig 15A). The addition of ubiquinol_10_ at concentrations of 10 and 20 μM immediately after UVA radiation at a dose of 10 J/cm^2^ significantly diminished the increased expression level of NEP protein at 72 h post-irradiation (Fig 15B). In contrast, the addition of RF at 0.2, 1 and 5 μg/ml did not elicit any significant abrogating effect on the increased expression of NEP protein (Fig 15C).

### Effects of ubiquinol_10_ and RF on the expression level of MMP-1 protein

**Fig 16:**
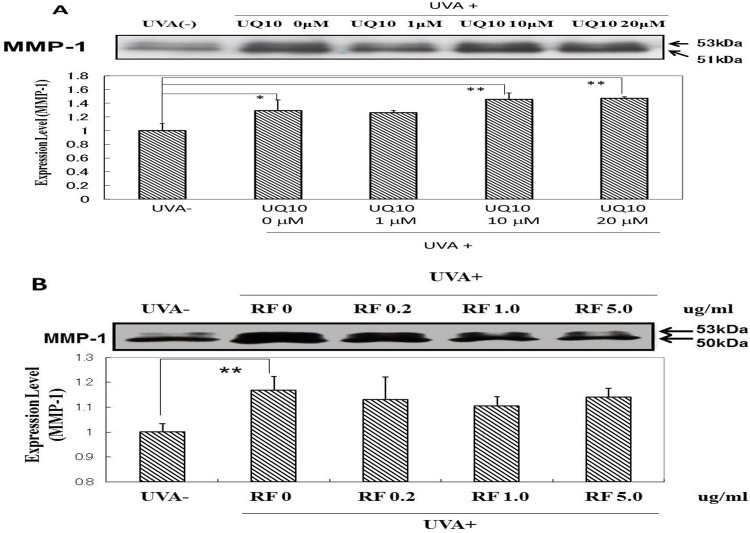
Effects of ubiquinol_10_ and RF on levels of MMP-1 protein expression in UVA-exposed HDFs. **A:** Treated with ubiquinol_10,_ **B:** Treated with RF**.** Levels of MMP-1 protein in the medium of UVA-exposed HDFs were assessed by Western blotting analysis at 72 h post-irradiation after treatment at the indicated concentrations. Equal μg protein was applied to SDS-PAGE. **UQ10:** ubiquinol_10_, **RF**: riboflavin, n = 3, *: p < 0.05, **: p<0.01.

When HDFs in culture were exposed to UVA at a dose of 10 J/cm^2^, the level of MMP-1 protein expression in the medium was significantly increased at 72 h post-irradiation (Fig 16). When ubiquinol_10_ was added at concentrations of 1, 10 and 20 μM immediately after UVA irradiation at 10 J/cm^2^, the significantly increased protein expression of MMP-1 at 72 h post-irradiation was further slightly up-regulated at concentrations of 10 and 20 μM ubiquinol_10_ (Fig 16A). On the other hand, the addition of RF at concentrations of 0.2, 1.0 and 5 μg/ml only induced a slight but not significant abrogating effect on the increased protein expression of MMP-1 at 72 post-irradiation (Fig 16B).

### Effects of ubiquinol_10_ and RF on NEP activity in UVA-exposed HDFs

**Fig 17:**
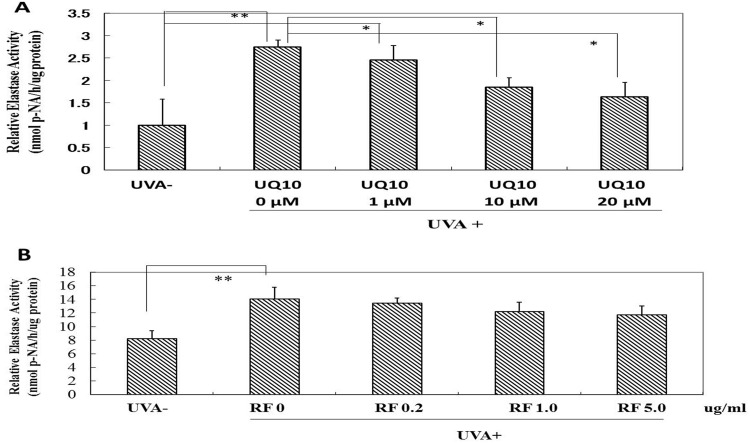
Effects of ubiquinol_10_ and RF on the NEP activity in UVA-exposed HDFs. **A:** Treated with ubiquinol_10,_ **B:** Treated with RF. The elastase activity levels were assessed by the enzymatic method described in **Material and Methods** at 72 h post-irradiation. **UQ10:** ubiquinol_10_, **RF:** riboflavin, n = 3, *: p < 0.05, **: p<0.01.

While UVA radiation at a dose of 10 J/cm^2^ induced a significant increase in the elastase activity, the addition of ubiquinol_10_ immediately after UVA radiation significantly diminished the increased activity of the elastase at all concentrations tested (1, 10 and 20 μM) (Fig 17A). In contrast, the addition of RF at 0.2, 1 and 5 μg/ml elicited a slight but not significant abrogating effect on the increased level of elastase activity (Fig 17B).

### Effects of ubiquinol_10_ and RF on MMP-1 activity in UVA-exposed HDFs

**Fig 18:**
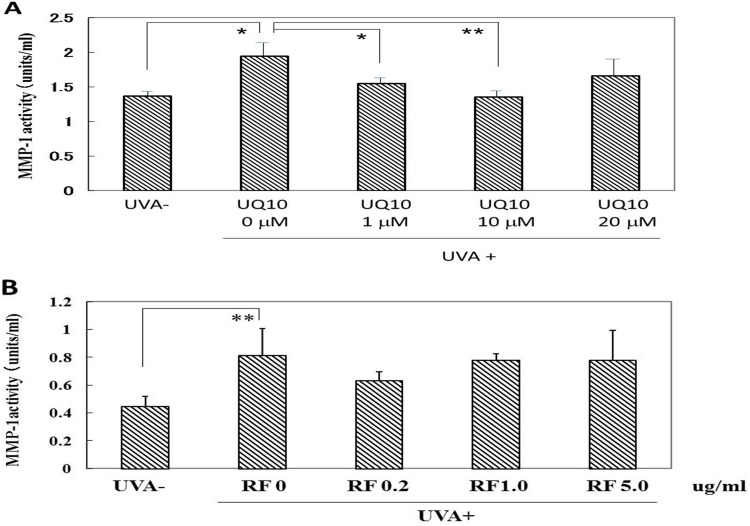
Effects of ubiquinol_10_ and RF on MMP-1 activity in the medium of UVA-exposed HDFs. **A:** Treated with ubiquinol_10,_ **B:** Treated with RF**.** Levels of MMP-1 activity in the medium were assessed by ELISA at 72 h post-irradiation. **UQ10:** ubiquinol_10_, **RF**: riboflavin, n = 3, *: p < 0.05, **: p<0.01.

While UVA irradiation at a dose of 10 J/cm^2^ induced a significant increase of MMP-1 activity in the medium of UVA-exposed HDFs, the addition of ubiquinol_10_ at concentrations of 1 and 10 μM immediately after UVA irradiation significantly abolished the increased MMP-1 activity at 72 h post-irradiation (Fig 18A). In contrast, the addition of RF at 0.2, 1 and 5 μg/ml did not elicit any significant abrogating effect on the increased level of MMP-1 activity at 72 h post-irradiation (Fig 18B).

### Secretion of cytokines in UVA-exposed HDFs and the effects of ubiquinol_10_ on the secretion

**Fig 19:**
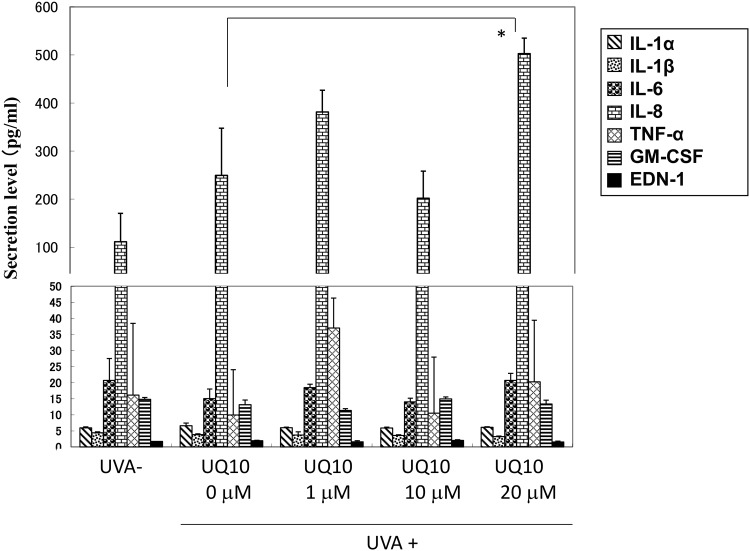
Secretion of cytokines by UVA-exposed HDFs and the effects of ubiquinol_10_ on that secretion at 48 h post-irradiation. The secretion levels of several cytokines in UVA-exposed HDFs at a dose of 10 J/cm^2^ were measured by ELISA at 48 h post-irradiation. n = 3, *: p < 0.05, UQ**10:** ubiquinol_10_, **IL-1α**: Interleukin-1α, **IL-1β:** Interleukin-1ß, **IL-6**: Interleukin-6, **IL-8:** Interleukin-8, **TNF**α: Tumor necrosis factor α, **GM-CSF**: granulocyte macrophage colony stimulatory factor, **EDN-1**: Endothelin-1.

Since the increased expression of NEP at the transcriptional, translational and enzymatic levels, and the increased activity of MMP1 were significantly abrogated by ubiquinol_10_ but not by RF, we next determined the effects of ubiquinol_10_ only on the secretion of various cytokines in UVA-exposed HDFs. When HDFs in culture were exposed to UVA at a dose of 10 J/cm^2^ and the cytokines released into the medium were measured for IL-1α, IL-1ß, IL-6, IL-8, TNFα, GM-CSF and EDN-1. The levels of cytokines released into the medium were not significantly changed for any of the cytokines tested at 48 h post-irradiation (Fig 19). The addition of ubiquinol_10_ at concentrations of 1, 10 and 20 μM immediately after UVA irradiation did not significantly change the released level of any cytokine tested at 48 h post-irradiation except for IL-8, the level of which was rather significantly up-regulated compared with UVA-irradiated and non-treated control (Fig 19).

## Discussion

Our long term studies concerning the biological mechanisms involved in UV-induced wrinkle formation [14, 15] have hypothesized that the over-expression of NEP, a specific matrix metallo-protease with elastase activity, by dermal fibroblasts in UV repeatedly-exposed skin plays an essential role in impairing the three-dimensional linear architecture of elastic fibers. That change in architecture leads to the loss of skin elasticity, which is distinctly associated as a major causative factor of wrinkle formation. Consistent with our previous studies [13, 16], the present *in vitro* study of the UV effects on NEP expression indicates that the markedly increased expression of NEP by dermal fibroblasts is mediated via biological actions triggered by the excess secretion of GM-CSF by UVB-exposed human keratinocytes as well as by human fibroblasts. The increased secretion of GM-CSF by HDFs is induced by IL-1α derived from UVB-exposed human keratinocytes and occurs at ~30-fold higher levels than those from UVB-exposed human keratinocytes. The results of the present study also indicate that direct UVA exposure of human fibroblasts significantly stimulates the expression of NEP at the protein and enzymatic levels without accentuating the secretion of GM-CSF. Thus, it is likely that the repeated exposure of human skin to natural sunlight stimulates the expression of NEP in dermal fibroblasts in two fashions; one mediated via UVB-associated epithelial-mesenchymal cytokine interactions and another occurring as a direct response to UVA.

Using a novel wrinkling model for UVB-associated epithelial-mesenchymal cytokine interactions, we found that all 4 anti-oxidants tested in this study interrupt the UVB-associated epithelial-mesenchymal cytokine interactions leading to the up-regulation of NEP in human fibroblasts but with different modes of action for each anti-oxidant. Thus, the above interrupting effect consists of at least two kinds of inhibitory mechanisms; one occurs via the inhibitory effect on UVB-stimulated secretion of two wrinkle-associated cytokines, IL-1α and GM-CSF, in human keratinocytes, and the other occurs via the abrogating effect on the IL-1α or GM-CSF-stimulated expression of NEP in human fibroblasts due to an interruption of the GM-CSF or IL-1α -activated intracellular signaling pathway as well as a direct inhibitory effect on NEP activity.

As for the first mechanism, we found that whereas AX and RF have a significant suppressive effect on both the increased secretion of IL-1α and GM-CSF, ubiquinol_10_ and FMN significantly abrogate the increased secretion of IL-1α, but not GM-CSF. Our finding that the conditioned medium from UVB-exposed keratinocytes triggers fibroblasts to remarkably accentuate the secretion of GM-CSF suggests that GM-CSF is a key intrinsic cytokine that causes fibroblasts to stimulate the expression of NEP in an autocrine and/or paracrine fashion. The evidence for the late gene expression evaluated by DNA microarray analysis and the secretion of GM-CSF relative to IL-1α in this study using UVB-exposed HPKs indicates that the increased secretion of GM-CSF is mediated via two different activations of NFkB pathways. Those pathways occur during both the direct UVB-activated and the IL-1α-stimulated signaling. Since both of those activated signaling cascades involve the translocation of NFkBp65 into nuclei and MSK1 activation, the latter of which is associated with the phosphorylation of NFkBp64Ser246 that serves as a stimulatory factor for DNA binding, our findings concerning the abrogating effects of inhibitors of NFkB translocation (JSH-23) and MSK1 activation (H89) on the increased secretion of GM-CSF are in good agreement with the above signaling mechanisms involved in the GM-CSF production.

Since all the anti-oxidants tested behave as scavengers for ROS generated by UVB irradiation, their possible inhibitory effects on the increased secretion of IL-1α and GM-CSF could be accounted for by the depletion of generated ROS if treated prior to irradiation. However, our findings that post-irradiation treatment with the anti-oxidants tested can also abrogate their increased secretion strongly suggest that they abrogate the up-regulated production and secretion of those factors via a novel signaling mechanism(s) in a ROS depletion-independent manner [25, 26] because the lifetime of ROS is very short (e.g. lifetime of •O2 is 4 μs) [24] except for H_2_O_2_ with 30 min life time. In UVB (20 mJ/cm^2^) exposed human keratinocytes, O_2_^-^ is predominantly formed, whereas peroxides including H_2_O_2_ and OH are slightly generated to a lesser extent [31]. Thus, it seems likely that antioxidants are not sufficient to deplete the predominantly generated ROS if treated immediately after UVB radiation. The observed abrogating effect on the UVB-stimulated secretion of IL-1α or GM-CSF in UVB-exposed keratinocytes even by post-irradiation treatment with all the anti-oxidants tested is substantiated by the evidence that post-irradiation treatments with anti-oxidants such as AX [25, 26] and oligomeric proanthocyanidin [27] can specifically inhibit the activation of mitogen and stress activated protein kinase (MSK)-1 in human keratinocytes and human melanocytes, respectively. Those inhibitions result in the attenuated phosphorylation of NFkBSer276, which leads to the abolishment of NFkB transcriptional activity [31, 32, 33, 34]. This MSK-1 inhibition by post-irradiation treatment occurs without any suppressive effect on the activation of the front line of stress activated signaling cascades such as p38/JNK/ERK, whose attenuation is generally evoked due to their ROS-depleting action if they are treated pre-irradiation [25, 26]. The inhibition of MSK1 results in the attenuated expression of NFkB-mediated genes through which many inflammatory cytokines including IL-1α and GM-CSF are mediated to increase their secretion levels in UVB-exposed mammalian cells [25, 26]. In UVB-exposed human keratinocytes, GM-CSF is secreted into the medium via an autocrine mechanism in which the secreted IL-1αstimulates the production and secretion of GM-CSF via the NFkB signaling pathway. We found in this study that the release of stored IL-1α due to UVB-induced cell membrane damage in the early phase of UVB irradiation is not detectable until 12 h post-irradiation, but in the late phase IL-1α undergoes de novo synthesis and is secreted into the medium at a detectable level at least until 24 h following UVB irradiation at a dose of 80 mJ/cm^2^. Since the observed secretion level of IL-1α at 72 h post-irradiation results from the de novo synthesis probably via the NFkB pathway, it is likely that all the anti-oxidants tested interrupt the de novo synthesis of IL-1α via a possible inhibitory effect on MSK1 activation during UVB-induced stress-activated signaling cascades leading to NFkB activation. As for the GM-CSF secretion pattern following UVB irradiation, we found in this study that the gene expression of GM-CSF is increased at least by 24 h post-irradiation. In addition, we have previously reported that the secretion of GM-CSF in UVB-exposed human keratinocytes is significantly increased by 20~24 h post-irradiation [13, 29]. Therefore, it is likely that the abrogating effects of AX and RF on the increased secretion of GM-CSF at 72 h post-irradiation are mediated via the interruption of NFkB activation including MSK1 activation which is directly induced by UVB radiation and/or is evoked by IL-1α released in an autocrine fashion. On the other hand, despite their interruption for the late phase of UVB-stimulated secretion of IL-1α, the failure of ubiquinol_10_ and FMN to abrogate the increased secretion of GM-CSF may be attributable to the lower levels of their inhibitory effects on the early released IL-1α triggered-activation of NF-kB cascades, especially MSK1 activation.

As for another mechanism of the inhibitory effects on the IL-1α or GM-CSF-stimulated activity of NEP, we found that ubiquinol_10_, RF and FMN, but not AX, elicited a significant abrogating effect on the IL-1α and GM-CSF-stimulated NEP activity. Since it is possible that the IL-1α and GM-CSF-stimulated activity of NEP is mediated via the activation of NFkB and MET receptor/MAPK signaling, respectively, in human fibroblasts, it seems reasonable to assume that ubiquinol_10_, RF and FMN have a distinct potential to interrupt these IL-1α or GM-CSF-activated signaling cascades probably by inhibiting MSK1 and Raf-1(up-stream target of MAPK) activation, respectively. This assumption is supported by our previous findings that some anti-oxidants can inhibit the activation of MSK1 during the UVB-induced activation of NFkB signaling in human keratinocytes [25, 26] and in human melanocytes [27] even by post-irradiation treatment. Thus, taken together, it is likely that the interrupting effects of ubiquinol_10_ and FMN on UVB-associated epithelial-mesenchymal cytokine interactions leading to the up-regulation of NEP in human fibroblasts are mediated via the combined inhibitory effects on UVB-stimulated secretion of IL-1α in keratinocytes and the GM-CSF-stimulated expression of NEP in fibroblasts. In contrast, the same interrupting effects of AX are mediated only via its distinct inhibitory effect on the UVB-stimulated secretion of IL-1α and GM-CSF. On the other hand, although its effect does not occur at a higher level, the same interrupting effect of RF is associated with its combined inhibitory effects on the UVB-stimulated secretion of IL-1α and GM-CSF in keratinocytes and the IL-1α or GM-CSF-stimulated expression of NEP in fibroblasts.

Using another wrinkling model for the UVA-stimulated expression of NEP in human fibroblasts, we found in this study that ubiquinol_10_ significantly abolishes the UVA-increased expression of NEP at the transcriptional, translational and enzymatic levels, whereas it does not suppress the UVA-increased expression of MMP-1 at those three expression levels. On the other hand, RF does not significantly abrogate the UVA-stimulated expression of NEP and MMP-1 at the transcriptional, translational and enzymatic levels except for the gene expression of MMP-1. Using the same model, we have already reported that the addition of FMN immediately after UVA exposure significantly attenuates the UVA-stimulated expression of MMP-1 and NEP at the gene, protein and enzymatic activity levels [17]. The addition of AX immediately after UVA exposure significantly abrogates the UVA-enhanced expression of MMP-1 and NEP at the gene, protein and enzymatic activity levels although both the UVA stimulation and subsequent AX inhibition are greater for MMP-1 than for NEP.

In conclusion, although whether or not the anti-oxidants tested in this study except for AX can affect MSK1 activation even by post-irradiation treatment remains to be determined, the sum of our findings strongly supports the *in vivo* anti-wrinkling effects of ubiquinol_10_ [20, 21] and AX [22, 23] on human and animal skin and provides convincing proof of the UV-induced wrinkling mechanism essentially focusing on the over-expression of NEP by dermal fibroblasts as an intrinsic causative factor [15].

## Abbreviations

ROS: reactive oxygen species
CoQ10: Coenzyme Q10
RF: riboflavin
FMN: Flavin Mononucleotide
AX: Astaxanthin
GM-CSF: granulocyte macrophage colony stimulatory factor
IL-1α: Interleukin-1α
NEP: neutral endopeptidase
STANA: N-succinyl-tri-alanyl-p-nitroaniline
UVB: ultraviolet B
